# Agro-ecosystem and socio-economic role of homegarden agroforestry in Jabithenan District, North-Western Ethiopia: implication for climate change adaptation

**DOI:** 10.1186/2193-1801-3-154

**Published:** 2014-03-21

**Authors:** Ewuketu Linger

**Affiliations:** Debremarkos University, Debremarkos, Ethiopia

**Keywords:** Non-tree based garden, Socio-economic role, Agro-ecological role, Climate change adaptation

## Abstract

Homegarden agroforestry is believed to be more diverse and provide multiple services for household than other monocropping system and this is due to the combination of crops, trees and livestock. The aim of this study was to assess socio-economic and agro-ecological role of homegardens in Jabithenan district, North-western Ethiopia. Two sites purposively and two villages randomly from each site were selected. Totally 96 households; in which 48 from homegarden agroforestry user and 48 from non-tree based garden user were selected for this study. Socio-economic data and potential economic and agro-ecosystem role of homegarden agroforestry over non-tree based garden were collected by using semi-structured and structured questionnaires to the households. Homegarden agroforestry significantly (P < 0.05) improved the farmers cash income than non-tree based garden. With insignificant garden size; homegarden agroforestry practice provides good socio-economical and agro-ecological service for farmers which have a higher implication for climate change adaptation than non-tree based garden.

## Introduction

### Background and justification

Large percentages of the Ethiopian population (80%) depend upon agriculture for their livelihoods, and contribute 42-45% of the total gross domestic product of the country (Zenebe *et al*. 2011). But currently the agricultural production falls under a risk due to a number of factors. Among them; climate change, land degradation in the form of soil erosion, soil fertility loss (which are important for grain yield production) and severe soil moisture stress, which is partly the result of loss of trees in their field and organic matter (Salvatore *et al*. 2011). Regarding climate change the country is one of world’s drought prone country, which lead to challenges in food production especially because 95% of the agricultural activity is dependent on rainfall (Eriksen and Kelly 2005). Rainfall variability and associated drought have been the major cause of food shortage and famine in Ethiopia (Salvatore *et al*. 2011). The whole effect of the above problem is loss of biodiversity, financial insecurity, food insecurity, subsequent increases in rates of malnutrition, which are becoming the major tribulations of human well-being, so that adaptation to this serious problem should be necessary.

Despite this, one of the solutions to meet diverse people’s requirement with fixed land is through the application of agroforestry which is more advantageous than monocropping (Mcneely and Schroth, 2006).

Homegarden with trees are one of agroforestry practices known to be ecologically sustainable and diversifies livelihood of local community. Homegarden is commonly defined as; land use system involving deliberate management of multipurpose trees and shrubs in intimate association with annual and perennial agricultural crops and invariably livestock within the compounds of individual houses, the whole tree-crop, and animal unit is being intensively managed by family labour (Kumar and Nair 2006). Here after, the term homegarden and homegarden agroforestry are used interchangeably throughout this paper.

The high diversity of species in homegarden have a wide socioeconomic and agro-ecological roles including production of food and a wide range of other products such as firewood, fodders, spices, medicinal plants and ornamentals (Unofia *et al.*^2012^) and avoidance of environmental deterioration of climate related hazards commonly associated with monoculture production systems (Fernandes and Nair 1990), income generating site (Shoo 2009). The diversity of plants in the homegarden associated with other organisms contribute to the formation and maintenance of soil structure, retention of moisture and nutrient levels and promotes the recycling of nutrients; which reduces ecosystem vulnerability to climate change (Verchot *et al*. 2007).

Despite its vast socio-economic importance, in Ethiopia the agro-ecosystem and socio-economic role of homegarden agroforestry are very few.

In order to maintain agro-ecosystem resilience and to meet the homegarden products for requirements of the people during stress of climatic hazard like drought, flood; scientific information is required. Lack of such scientific knowledge of homegarden agroforestry may let destruction of plant diversity and results soil erosion during high rainfall, less income, food insecurity and hunger during drought period. Without a full assessment of socio economic and agro-ecosystem benefit as compared to non-tree based garden, their relation to adapt the changing climate and their role for rural poor people cannot be fully explained. In this particular research non-tree based garden refers to growing of only cereal/cash crop component (maize, teff, barley, wheat, pepper) each of them grown alone or mixed in a household garden rarely with boundary tree component. Therefore this research is aimed at I) Assess the socio-economic role of homegarden agroforestry in comparison to non-tree based garden in Jabithenan district. II) Assess agro-ecosystem role of homegarden agroforestry in comparison to non-tree based garden in Jabithenan district.

## Materials and methods

### Description of the study area

The study was conducted in Jabithenan district, North-western Ethiopia. Geographically the district is found at 10°40′northern latitude and 37°11′eastern longitude.

The topography of the district is generally characterized by flat gentle slope (65%), mountainous (15%), undulating terrain (15%) and valley (5%), with an altitudinal range from 1500 - 2300 m a.s.l. The major soil types found in the district are Vertisol and Nitosol (JWARDO Jabithenan Woreda Agriculture and Rural Development Office 2012).

Climatically, the district falls within midland and lowland agro-ecological zone. The mean annual temperature is about 23°C, with a maximum temperature slightly above 32°C, down to a minimum of 14°C. The mean annual rainfall ranges between 800 – 1250 mm.

The total population of the district is 277,590, of whom 139,616 are males and 137,974 are females. Of this total population, 6% of their population is urban dwellers and the rest 94% are rural dwellers. An estimated population density of the district is about 455.32 people per square kilometers (JWARDO Jabithenan Woreda Agriculture and Rural Development Office 2012).

Historically, the district was covered by dense natural forests, but the distribution of natural forest is declining from time to time, owing to human interference. The common vegetation in the district include, *Croton macrostachys, Ficus sur, Albizia gummifera, Cordia africana, Acacia abyssinica, Rosa abyssinica* and *Erythrina abyssinica* which are found as scattered in most farm lands. While *Eucalyptus* spp and *Gravillea robusta* are grown as boundaries, live fences and woodlots (JWARDO Jabithenan Woreda Agriculture and Rural Development Office 2012).

Agriculture is the principal source of livelihood for rural population. It is characterized by subsistence mixed farming of rain-fed, irrigated crops, and livestock production together with trees planted as an agroforestry. In the district cereal crops are the staple food crops such as teff (*Eragrostic teff*), maize (*Zea mays*), wheat (*Triticum sativum)* and barley *(Hordeum vulgare)* are the most commonly cultivated crops. Pepper (*Capsicum frutescens)*, sugarcane (*Saccharum officinarum)*, coffee (*Coffea arabica*) and chat (*Catha edulis*) are the dominant cash crops in some sites including the study village (JWARDO Jabithenan Woreda Agriculture and Rural Development Office 2012).

### Sampling method

Purposive sampling method was employed. Two sites (here after Kebele Administrations^a^, KA) namely, Mankusa Abdegoma and Jiga Yelmdar were selected purposefully based on the extensive presence of both homegarden agroforestry (HGAF) and non-tree based garden (NTBG). Potential villages (five villages from Mankusa Abdegoma and three villages from Jiga Yelmdar Keble) were identified by reconnaissance survey with district and Kebele agricultural office experts. Then Debohela and Waza villages from Mankusa Abdegoma KA; Atahagne and Tikurwuha villages from Jiga Yelmdar KA were randomly selected.

In this study, key informants and households were involved to assess how HGAF is important to them over NTBG. KIs for this study is defined as persons who are knowledgeable about the previous and current situation of local climate conditions and role of various HGAF for climate change adaptation, agro-ecosystem maintenances and who lived there at least for continuous 30 years. KIs were selected by snowball method (Bernard 2002). Accordingly during village reconnaissance, five farmers were randomly asked to give the name of six KIs. Out of the mentioned thirty candidate KIs, the five top ranking were selected at each village which make a total of 20 KIs for the entire study. Those selected KIs with DA were also used to classify household into two garden types and wealth categories based on local wealth criteria. HH is defined as a basic unit of production and consumption, made up from the persons who manage common landholdings and live less than one central decision-maker, the household head. Finally, from each wealth class 8 HHs were picked randomly (4 HGAF and 4 NTBG practicing HH), making 24 HHs per village and 96 HHs for the entire study.

### Data collection

Based on information from KI interview, questionnaires were designed to collect data on the role of gardens in socio-economic and agro-ecosystem maintenance and associated implication when climate variability/change occurs was collected. Focused group discussions were also used to validate the information given by an individual farmer and to catch important issues that were not raised by respondent farmers.

Two years (2011/2012 and 2012/2013) HH income from two garden types was collected. Product from HGAF that were consumed by household as food were not converted in to cash, since in every time children’s are using especially fruits and tubers of herbs for food and farmers are unable to determine the exact value. Crops produced from NTBG were converted in to cash according to the current market during data collection time. Both socio-economic and inventory data were collected from early November to end of December 2012 while garden income of the year 2012/13 were collected in one month (April 2013).

### Data analysis

Data collected from the interview and questionnaire was coded, enters in to a computer, analyzed, interpreted and synthesized using Statistical Package for Social Sciences (SPSS) software Version 16. Socio-economic and agro-ecosystem role between garden types was subjected to one-way ANOVA. Mean differences between groups were considered significant at p <0.05.

## Results

### General evidence of climate variability in the study village

**Figure 1:**
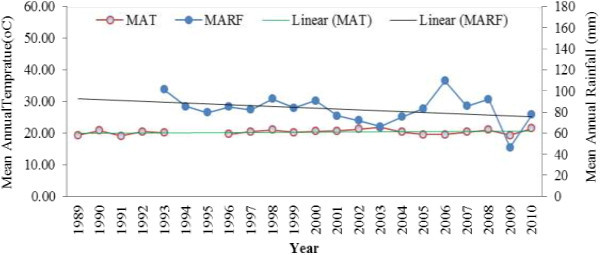
**Mean Annual rainfall and temperature from 1989-2010 in Mankusa Abdegoma Kebele, Jabithenan district, Ethiopia.**

**Figure 2:**
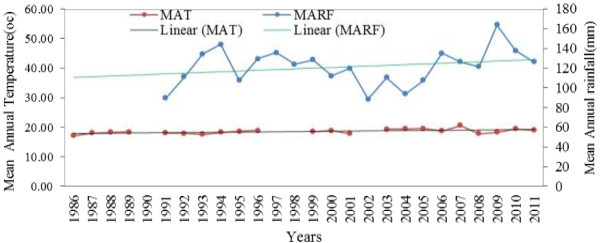
**Mean annual rainfall and temperature from 1986-2011 in Jiga Yelmdar Kebele, Jabithenan district, Ethiopia.**

Particularly rainfall and temperature for the study kebeles were taken from metreology agency of the nearby station (Figures 1 and 2). For Mankusa Abdegoma Kebele^b^ lay-ber station was the source which is 10 km far from the study Kebele. Whereas for Jiga Yelmdar, Yechereka station was used having 6 km distance (NMA National Metreology Agency 2012). MAT^c^ and MARF^d^ were used.

### Socio-economic characteristics of respondents

Household’s socioeconomic features can influence on-farm/garden tree selection, management and species diversity. In this study, of the 96 participating households, 78% were male headed whereas the remaining 22% were female headed. Number of dependent ranged from 1 to7 person per HHs and the number of labor force ranged from 2 to 9 across all villages. Education levels of a HH have also a direct influence on the management of agroforestry practices or in adoption of new technology. Of total respondent HHs 64%, 21%, 10%, and 5% were illiterate; attended Grade 1-4, 5-8 and secondary school respectively.

### General features of garden types

#### Homegarden agroforestry (HGAF)

The original layout of most gardens was established during the villagization program of the military (Derg) regime (1974-1992). Then after producing cereal crops for some years, in 1992 about19%, in1995 (67%), and 14% of households in 1996 were integrate perennial trees in their garden and becomes HGAF (n = 48).

Spatial arrangement of HGAF is variable at the study site. About 74% of HGAF is located in the backyard while a few are located on the side of the homestead. Most of the studied HGAF (67%; n = 48) are surrounded by live fence of the species *Eucalyptus camaldulenesis*, *Capparis tomentosa, Rosa abyssinica, Carissa edulis, Combretum molle, Maesa lanceolata, Bersama abyssinica, Acacia nilotica*. The rest 33% are semi-fenced and open.

The overall HGAF size ranged from 0.05 to 0.5 ha (0.1 to 0.5 ha in Jiga Yelmdar and 0.05 to 0.38 ha in Mankusa Abdegoma Kebele). The average size of HGAF in Jiga Yelmdar and Mankusa Abdegoma Kebele is 0.21 ha and 0.16 ha respectively. Most of the surveyed HGAF (63%) in Mankusa Abdegoma and (60%) in Atahagne village of Jiga Yelmdar kebeles have rectangular shape while the remaining were irregular and square shape.

#### Non-tree based garden (NTBG)

**Figure 3:**
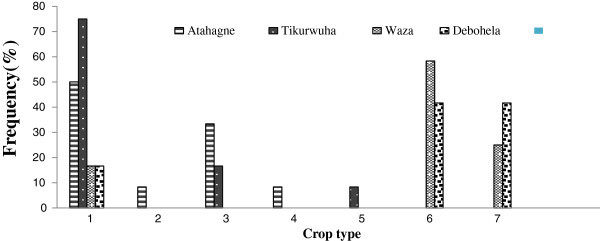
**Types of crops produced in non-tree based garden in year 2012 and proportion of respondent households at four villages, Jabithenan district, Ethiopia.**

**Figure 4:**
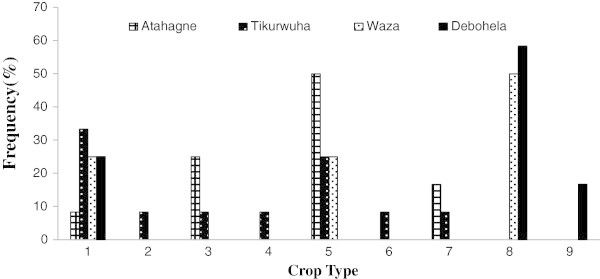
**Types of crops produced in non-tree based garden in year 2013 and proportion of respondent households at four villages, Jabithenan district, Ethiopia.**

Like HGAF, the overall NTBG size ranges from 0.05 to 0.5 ha. But in contrary, NTBG were covered by cereal/cash crop (Figures 3 and 4). From the 48 surveyed NTBG, 7 numbers of species (i.e. species richness) were recorded. Those species are Maize (*Zea mays*), Potato (*Solanum tuberosum)*, Wheat (*Triticum sativum*), Barely (*Hordeum vulgare*), Sugarcane (*Saccharum officinarum)*, and Onion (*Allium cepa)* and pepper (*Capsicum frutescens*). Figures 3 and 4 indicates the frequency of cereal crop recoded out of the 48 interview respondents households in two successive years at each village. Onion, pepper and sugarcane are an important source of cash for the household.

### Advantage of homegarden agroforestry over Non-tree based garden

HGAF plays an important role and unique land management system because of the potential role in addressing biophysical, economical and socio-ecological components. Such diversity and interaction leads to greater functional and structural complexity as compared to non-tree based garden. Surveyed households admire homegarden importance especially during climate related shock happen. Farmers grouped climate related hazards in to three major topics: climate variability (100), drought (87), rain with ice (92) which hampers production of cereal crops. The value in the bracket shows respondents percentage.

#### Socio-economic role

Economic difference was great among the two garden types with almost proportional social group (religion, age group, work habit, land size, homegarden size), infrastructure and extension service. The focused group discussions employed also indicated that production of cereals in NTBG is limited to only rainy season (Table 1).

**Table 1:** **Summarized characteristics of HGAF and NTBG at four villages, Jabithenan district, Ethiopia**

	Garden Type	
	HGAF	NTBG
**Major characteristics and use for the household**	High diverse species	Less diverse species
	Production is throughout the year	Production is once, rarely 2 times (irrigation)
	Saves working time during peak labor work month through provision of immediate fresh food	Households wait until the food is cook, time wasted
	High labor cost	Less labor cost
	Reduce cost of purchasing fertilizer	Use chemical fertilizers ( Incurs cost)
	Increase social intimacy through coffee ceremony	Involvement in coffee ceremony is limited to relatively well-off HHs.
	Use of fruits reduce frequency of request for meals and avoid hunger especially for children	Relatively higher frequency of request for meals 3 to 5 times/day

The study revealed that, different species grown in the homegarden gives different functional roles for the household. In HGAF, 77 and 67% of respondent consume fruit trees as food and generate cash income respectively. HGAF also provide fuel wood much greater than NTBG, which is the major energy source in the site. In addition to home use, some households get an income from fuel wood selling and 19% of respondent farmers also prepare charcoal from *Acacia abyssinica.*

Households in the study area generate income from different homegarden products. Income results from two garden type shows significant difference (Table 2).

**Table 2:** **Mean farm size, garden size, annual income of 2011/12 and 2012/13 from the two land use types of four villages, Jabithenan district, Ethiopia**

Village	Garden type	Farm size(ha)	Garden size(ha)	Cash income (Birr) 1$ = 19birr when the research conducts	
				Year 2011/12	Year 2012/13
Tikurwuha	NTBG	1.67a ± 0.16	0.25a ± 0.03	2,266.67aA ±349.31	1,612.50aA ± 351.73
	HGAF	1.69a ± 0.18	0.22a ± 0.02	15,750.00bB ± 3735.83	10,050.0bB ± 3345.54
Atahagne	NTBG	1.77b ± 0.20	0.17b ± 0.03	1,867.33cC ± 308.06	1,353cC ±210.27
	HGAF	2.15b ± 0.24	0.23b ± 0.03	22,483.33dD ±9361.23	13,112dD ± 4351.57
Debohela	NTBG	1.17 c ± 0.09	0.14c ± 0.02	5,825.00eE ±1354.46	2,487.5eE ± 905.71
	HGAF	1.13c ± 0.10	0.10c ± 0.01	6,858.33eF ± 1549	5,857.5eF ± 1914.10
Waza	NTBG	1.00d ± 0.09	0.18d ± 0.03	5,987.50fG ±1716.81	770.00fH ± 149.21
	HGAF	10.21d ± 0.10	0.14d ± 0.02	6,945.83fI ± 1609.72	7,033.33gI ± 2298.40
Overall mean	NTBG	1.40 ± 0.14	0.19 ± 0.03	3,986.50 ± 932.16	1,555.63 ± 404.23
	HGAF	1.55 ± 0.16	0.17 ± 0.02	13,009 ± 4063.95	9,013.21 ± 2977.40

NB - Single different small letters on mean values indicate significant difference at (P < 0.05) between the two land users with in the village in a column.

Single different capital letters on mean values indicate significant difference of income at (P < 0.05) between the two years with in the same land use in the village horizontally (row).

#### Agro-ecosystem role

HGAF play an important role for agro-ecosystem service mainly through providing raw material for compost production (Table 3). Without significant difference in the demographic character and land or homegarden size between garden types (Table 2), 100% of the respondent confirms that the different in the volume of compost produced was due to the raw material (weeds and grasses, tree leafs) available within a HGAF. Using HGAF for compost production 94% of the respondents (n = 48) say that, the fertility status of the soil stays up to a minimum of three years and a maximum of four years in addition to soil moisture conservation while rainfall shortage happens (Table 4).

**Table 3:** **Mean volume of compost and number of livestock/household in the two garden types at four villages, Jabithenan district, Ethiopia**

Village	Garden type	Volume of compost (m3)	Number of livestock
Atahagne	HGAF	23.67a ± 4.18	14a ± 1.94
	NTBG	9.92b ± 0.99	7b ± 0.84
Tikurwuha	HGAF	25.5a ± 3.01	16a ± 2.34
	NTBG	9.08b ± 1.27	6b ± 0.84
Waza	HGAF	16.58a ± 3.29	7a ± 1.14
	NTBG	9.33a ± 2.17	12a ± 1.79
Debohela	HGAF	21.58a ± 2.97	7a ± 1.40
	NTBG	7.64b ± 2.13	5a ± 0.47
Overall mean	HGAF	21.83 ± 3.37	11 ± 1.70
	NTBG	8.99 ± 1.64	8 ± 0.99

NB - Single different letters on mean values indicate significant difference at (P < 0.05) between garden types in a village in a column.

**Table 4:** **Percentage response of two garden type users’ on gardens ecosystem role at four villages, Jabithenan district, Ethiopia**

Parameters	Percent response	
	HGAF (n = 48)	NTBG (n = 48)
Inorganic fertilizer application in garden	0	81
Herbicide/pesticide application in the garden	0	87
Presence of erosion in garden	0	92
Use of animal dung for soil fertility	77	12
Crop residual removal from outside garden	40	92
Presence of moisture during rainfall shortage	94	15
Garden fodder tree increase livestock number	71	0
Compost reduce fertilizer coast	85	35

HGAF provide fuel wood source which leads to less farm crop residual biomass removal, less dependence on animal dung for fuel and minimum investment of money for organic fertilizer than NTBG (Table 4).

## Discussion

### Homegarden agroforestry: contribution and implication to climate variability/change adaptation

The species in the NTBG was composed of mainly cereals and their coverage/type of species varies in time/year. In 2011/2012 onion was not recorded; but was planted in year 2012/2013 during winter through irrigation. The reason is because as 100% of respondent confirms, there was seasonal (three weeks) rainfall shortage (drought), crops were failed due to less water availability during growing season in 2012/13. Even in the beginning, onset of rain was late (June 22, 2012) and lags from the normal rainfall year (early may), which hampers crop production. Then NTBG users immediately react/cope by doing income generation activity basically onion irrigation from the distance water, which is labor intensive. Since HGAF has diverse component than NTBG, risk was lower in HGAF and have the following role.

#### Food and income role

Species in the studied HGAF supplement for household’s food and income. Tubers and vegetables are among the herb species and replace the staple food of cereal crop. Research results also showed that tubers are replacing the basic staples of cereals, and produce reasonable amount of carbohydrate (Fernandes and Nair 1986). Respondents assure that fruit trees are primarily served for food especially during difficult time of drought. The three fruit tree species frequently found was an indicator of how farmers are highly depends in HGAF for food in addition to their cash income revenue. Relatively *Persea americana* and *Mangifera indica* have a good market (but not consistent) in the near zonal town, Fintoselam than other fruit tree species. This is due to the fact that urban peoples use the processed juice from the two fruit tree species. Fentahun Mengistu (2008) also confirms, fruit trees from HGAF have significant role during environmental crisis of households. While family/especially children’s consume fruit trees, it is possible to avoid (buffer) frequency of hanger and decreases number of meals/day. So dependency on other cereal food crops becomes minimal. In the long run, cereal crops are reserved/saved to buffer households during time of stress. In contrary, in NTBG land user this is not possible since household members have no other substitution food items to save cereal crops for difficult time, and so the vulnerability becomes higher than HGAF user. Research findings from South Africa agroforestry fruit trees show, fruits play an important role especially during time of famine and other stress as food, nutrition and cash income (Akinnifesi *et al.*^2008^). All other components of homegarden in the study area help the same as what fruit tree does in one way or other. According to Scherr (1995), rural households survival strategies encompass multiple objectives in maximization of utility, like provision of food, and subsistence goods, cash for purchase of goods, services and saving for future needs, while environmental or other shock happens; in which HGAF can do. A study in Zambia showed that some products from homegardens, particularly fruit and cabbage are important buffers during drought years (Alfred 2009) and consistent with this study.

Income result between the garden types in Jiga Yelmdar was significant, unlike Mankusa Abdegoma Kebele in 2011/2012. Sugar cane and pepper both a high value marketable crop were dominantly occurred in Mankusa Abdegoma.

In 2012/2013 income result from Jiga Yelmdar kebeles was significantly higher in HGAF than NTBG. This explanation could be due to the double factors (drought and less value market crops in NTBG).

In 2012/2013 income results from Waza village of Mankusa Abdegoma kebeles was significantly (P < 0.05) higher in HGAF. Even NTBG were covered by high marketable crops, insufficient moisture content contribute to the reduction in cash income. In this village the income was not significantly vary in 2011/2012, since no drought year. A study in Amhara region, Ethiopia showed that production of cereal crops (wheat, barely, maize, teff, sorghum) showed statistically significant correlation with seasonal rainfall variability during 1994-2003 (Bewuket 2009).

Current research Journals confirmed that, relaying in HGAF remains viable for foreseeable future as a strategy to stabilize household food security and income against the risks uncertainties of monocropping due to multiple products (Alfred 2009). Other findings also confirm that HGAF contributes to household risk management via reducing income variability (FAO Food and agricultural organization 2011).

The sale of products from homegarden significantly improves family financial status, and cash income can be used to buy food, cloth etc. This observation is in line with the study done in homegarden of Zimbabwe and Ethiopia (Alfred 2009; Kebebe and Urgessa 2011).

The presence of fodder tree has also a crucial value, increases the number of livestock (important assets during time of crisis). Review report from dry land of Africa showed that presence of fodder tree in the garden serves not only increase number of livestock but also reduces livestock forage cost (Bashir *et al*. 2006). Other findings also show that an increase in number of livestock leads to an increase in food stock due to livestock waste is useful in the farming to improve crop yield (Anne 2008).

#### Ecological role

The multi-layered, forest like vegetation structure of the studied homegarden in the area contributes substantially to the agro-ecological sustainability through reducing soil erosion. Research findings from homegarden of Meghalaya, North-east India also confirms that, multilayered vegetation structure prevents soil erosion, provides habitat to soil micro-organisms and promote a favorable microclimate for the household (Tynsong and Tiwari 2010). Fernandes and Nair (1986) also reported that the presence of multi-layered structure of homegarden is an indicator of ecological function through environmental protection and efficient use of resource (like sunlight).

HGAF play a role not only erosion control in the garden, but also give three or four year soil fertility maintenance out of the garden/other farm field and saves soil moisture through composting. Compost is not only give soil fertility and moisture conservation, but also reduces the farmer’s fertilizer cost (could be invested in other economic activity like rent land to produce another crop). Alfred 2009 cited in Chivaura-Mususa *et al.* (2000) showed that livestock manure, litter fall and compost was found a rich in nitrogen and becomes a good fertilizer to maintain soil fertility and crop production. The result obtained here is also in line with other findings in homegardens of Wayanad, Kerala; homegarden supply raw materials (such as leaf and compost) to agricultural land for the local community (Santhoshkumar and Lchikawa 2010). Bishaw *et al.* (2013) reported that the higher number of livestock due to fodder tree availability provide significant amount of manure that can go in to improving soil fertility. HGAF give fuel wood; reduce pressure on animal dung. So farmers of HGAF user were applying this dung for soil fertility. Whereas majority of NTBG, animal dung were used for both fuel wood and soil fertility; and purely for fuel wood.

HGAF also helps for crop residuals to be remaining there in the farm (since HGAF serves what crop residual serves) after the grain harvest. Residuals in the process decompose and help for soil fertility. In NTBG majority of the respondent harvests residuals for livestock feed and fuel wood, which reduce soil fertility. Therefore, the overall soil fertility maintenance, soil moisture conservation help the crops to be grown, not to wilt when rain fall variability happens for one/two weeks.

#### Social role

Some households gave some homegarden products (like fruits, vegetables, sugarcane) to neighbors and relatives, which strengths nebiour and family relationship called social capital. When there is a religious day (e.g. Saint Gebreal and Micael), rural peoples have a coffee ceremony in circulation with their neibour. But a person/family who has not coffee in his garden was not participating in the ceremony, since he/she can’t able to return in other days. NTBG users participate, if they are able to purchase coffee from the market. In this case coffee as a HGAF component increase social relationship and sharing different working experience (regarding homegarden species management, harvest) from model farmers, unlike NTBG users. This observation is in consistent with the results from research in Zambia, homegarden of Meitei community of India (Alfred 2009; Davi and Das 2010) helps for good social relation through sharing of homegarden vegetables. The overall implication is increasing intimacy and farmers can help each other when a farmer/household becomes food insecure. Researchers from social science agree that, social capital reduces/adapt risks of climate change; avoid conflict through interaction between people or state (Neil 2003; Bishaw *et al*. 2013).

## Conclusion and recommendation

The associated component diversity of Homegarden agroforestry enhances the livelihood of the local people by providing socio-economic and agro-ecological service than non-tree based garden; important to adapt climate change or climate related stress (drought).

The government should work jointly with the local farmers in changing monocrop land use system to mixed agroforestry system for climate change adaptation.

## Endnotes

^a^KA is the lowest administrative unit in Ethiopian government structure.

^b^Kebele is the lowest administrative unit in Ethiopia.

^c^MAT, Mean annual temperature (mean of 12 month temperature in a year).

^d^MARF, Mean annual rain fall (mean of 12 month rainfall in a year).
